# Physical activity phenotypes in endometriosis using unsupervised learning via functional mixture models

**DOI:** 10.1186/s12905-025-04087-2

**Published:** 2025-12-11

**Authors:** Bryan T. Tricoche, Billy A. Caceres, Leslee J. Shaw, Carol Ewing Garber, Stefan Konigorski, Sahiti Kolli, Thomas J. Fuchs, Ipek Ensari

**Affiliations:** 1 Windreich Department of Artificial Intelligence and Human Health, Icahn School of Medicine at Mount Sinai, New York, NY USA; 2 Columbia University School of Nursing, New York, NY USA; 3 Department of Medicine (Cardiology), Population Science and Policy, Obstetrics, Gynecology, and Reproductive Science, Icahn School of Medicine at Mount Sinai, New York, NY USA; 4 Blavatnik Family Women’s Health Research Institute, Icahn School of Medicine at Mount Sinai, New York, NY USA; 5 Department of Biobehavioral Sciences, Columbia University Teachers College, New York, NY USA; 6 Hasso Plattner Institute for Digital Engineering, University of Potsdam, Potsdam, Germany; 7 Hasso Plattner Institute for Digital Health at Mount Sinai, Icahn School of Medicine at Mount Sinai, New York, NY USA

**Keywords:** Endometriosis, Physical activity, Functional data analysis, Unsupervised learning, Functional mixture models, Wearable devices, Fitbit, Symptom management, Pain, Fatigue

## Abstract

**Background:**

Endometriosis is a chronic condition associated with severe pelvic pain, dysmenorrhea, infertility, and worsening quality of life. Regular physical activity (PA) is effective for pain management and reducing chronic disease symptoms, yet individuals with endometriosis are more likely to be insufficiently active. This study investigated latent profiles of daily PA trajectories in this population via clustering.

**Methods:**

We analyzed 171 adults (4,795 person-level days) with a confirmed diagnosis of endometriosis enrolled in the *All of Us Research Program*. PA data were collected from participants using Fitbit wrist-worn trackers. We used 30 consecutive days of data from each individual, allowing up to 10 days of missingness, imputed using multiple imputed chained equations. Functional mixture models (FMMs) were used to identify latent PA trajectory clusters using daily step counts as the outcome variable. The optimal number of clusters was selected via Bayesian Information Criterion (BIC). Exploratory analyses of PROMIS pain and fatigue surveys were conducted in a subset of 129 participants who completed the surveys after their PA time windows.

**Results:**

FMM-identified profiles differed both with respect to PA volume and variability. Combinatory model fit indices supported a 4-cluster (K = 4) solution. The *“*High Active*”* phenotype exhibited the highest volume and variability of daily step counts and moderate-to-vigorous PA (MVPA) minutes over the sampling period (Steps: Mean (SD) = 12918.8 (5606.4); MVPA: Mean (SD) = 75.2 (64.6)). The *“*High Moderate*”* phenotype exhibited the second highest activity (Steps = 9283.9 (3661.2); MVPA = 58.2 (59.6)), followed by *“*Low Moderate*”* (Steps = 6234.0 (2515.8); MVPA = 18.6 (32.3)), and *“*Insufficiently Active*”* (Steps = 4317.1; MVPA = 17.2 (28.9)). Exploratory analyses revealed that higher-activity phenotypes tended to report lower pain scores. However, the “High Active” phenotype had the highest proportion of individuals reporting severe to moderate fatigue.

**Conclusion:**

This is the first study to investigate and report distinct PA profiles among a nationally-representative sample of individuals living with endometriosis using objectively-estimated PA. Identifying phenotypes based on within- and between-individual variance may help identify those at risk and inform the development of personalized interventions aimed at promoting PA and improving health outcomes in this population.

**Supplementary Information:**

The online version contains supplementary material available at 10.1186/s12905-025-04087-2.

## Background

Endometriosis is a chronic, estrogen-mediated, inflammatory condition characterized by the growth of endometrial-like tissue outside the uterus, leading to the formation of adhesions and lesions. This condition affects approximately 10% of women of reproductive age and is associated with severe pelvic pain, fatigue, dysmenorrhea, and infertility, significantly reducing the quality of life (QoL) of affected individuals [1, 2].

Regular physical activity (PA) has been recognized as a crucial factor in maintaining overall health and reducing the risk of non-communicable diseases. While engaging in regular, sufficient PA might be impeded by an individual’s chronic disease [3, 4] (e.g., endometriosis pain), PA has been demonstrated to be beneficial for managing endometriosis symptoms, including pain reduction and improved QoL [3, 5–7]. Cross-sectional studies [6, 8] investigating the relationship between PA and endometriosis symptoms found that women who engaged in more PA reported lower pain levels and better QoL.

Existing cohort-based studies in people living with endometriosis mostly rely on self-reported measures, cross-sectional sampling, or tools that are not comprehensive or validated for assessing PA [9]. Self-reported measures can be subject to recall bias and other sources of measurement error [10], thus limiting the quality of the data and the inferences made from them. Objectively-estimated longitudinal data toward this end can provide a more accurate and comprehensive picture of PA patterns in this population, as well as longitudinal patterns over time [11, 12]. These data can subsequently help clinicians identify those who don’t meet PA guidelines and those who can benefit from tailored PA interventions.

Traditional approaches to analyzing PA data typically involve aggregating multiple data points per person to get a summary score (e.g., daily or weekly PA volume). This approach, however, does not capture temporal trends and patterns over time, which could provide additional valuable information about activity patterns within a study population [12]. Among individuals with endometriosis, characterizing the temporal and between-individual variations in PA patterns can help identify potentially distinct PA profiles. This endeavor remains to be explored using wearable-derived PA data and a functional data framework via mixture modeling, which can yield more informative findings regarding the temporality of PA behavior. These phenotypes can then be used to tailor PA recommendations and support strategies to each patient’s specific activity patterns and needs. This personalized approach may lead to more effective management of endometriosis symptoms and improved overall health outcomes. In addition, consideration of temporal patterns can help assess the effectiveness of PA interventions by examining changes in activity profiles over time, as well as serve as early indicators of symptom flare-ups or disease progression.

The present study investigates latent profiles of daily PA trajectories among individuals with endometriosis using functional mixture models (FMMs). FMMs consider entire data curves as the unit of analysis, as opposed to discrete data points, making them more flexible and robust for examining the shape of the PA trajectory over time.

Accordingly, the primary aim of this study is to identify potential phenotypes of PA patterns among individuals living with endometriosis. We use the *All of Us Research Program* cohort to conduct our analyses, which provides a sample of participants across the United States (U.S.). Given the literature on the symptomatic heterogeneity in this population, we hypothesized that there would be distinct, latent clusters (i.e., phenotypes) based on varying PA patterns with respect to daily volume and variability [13]. We expected that the identified phenotypes would differ significantly based on temporal fluctuations in PA, with higher volumes of PA being associated with greater variability.

We further complement the phenotype characterization by assessing symptom burden based on the Patient-Reported Outcomes Measurement Information System (PROMIS) questionnaires on pain and fatigue [14–16]. These exploratory analyses examine whether symptom severity varies across phenotypes and serves as preliminary insight into how subjective experiences of endometriosis relate to objectively-estimated PA patterns.

## Methods

### Study sample

The study sample included adults with a confirmed diagnosis of endometriosis enrolled in the NIH *All of Us Research Program*. A detailed description of the research program has been published elsewhere [17, 18]. We used the *All of Us* Registered Tier Dataset v6, which was made available in June 2022, for our analysis [19]. The *All of Us* Researcher Workbench is a secure cloud-based platform that allows approved researchers to use Jupyter Notebooks to access the data of participants that have consented to share their data within the *All of Us Research Program’s* Curated Data Repository (CDR) [19].

Inclusion criteria for the analytic sample were as follows: (1) Being 18 + years of age at the time of data collection; (2) Having an endometriosis diagnosis based on either electronic health record (EHR) data OR self-reported surveys (“*Has a doctor or health care provider ever told you that you have endometriosis?*”), which enable inclusion of a comprehensive cohort [16]. This was also done because EHR data might not have been available for participants in the *All of Us Research Program;* and (3) Having available Fitbit data on PA within the *All of Us* dataset. Upon enrollment, participants have the option to give permission to link their Fitbit devices with the Workbench, which allows use of their PA data from their trackers. The analytic sample therefore is limited to those for whom such data were available. While endometriosis is typically diagnosed during reproductive years, the duration of its impact can vary across individuals. We therefore did not limit the upper age to allow extension into older age groups and enhance the age diversity in the sample. All participants provided written informed consent to enroll in the *All of Us Research Program*.

### Study variables

#### Outcome variable

We used daily step counts as the outcome variable based on several reasons.

First, step counts capture all intensities and ambulatory types of PA, therefore providing the most comprehensive measure of habitual PA as per our *a priori* goal. Second, there are published cut-points for step counts that have been linked to overall health outcomes, which allows their evaluation as a clinically meaningful, intuitive PA metric [20–22]. Third, step counts are the most frequently used PA parameter in literature [23–25], which allows for easier comparisons across studies, although such comparisons should be made cautiously as step count algorithms may differ by device and study.

#### Other PA variables

To characterize the model-identified phenotypes’ PA volume, we used light-, moderate-, vigorous-, and combined moderate-to-vigorous physical activity (MVPA) minutes recorded from wrist-worn Fitbit devices [26, 27]. MVPA was calculated by adding the moderate and vigorous intensity PA minutes. Fitbit calculates light, moderate, and vigorous intensity PA minutes using its proprietary algorithms, which integrate accelerometer motion data, heart rate, and age [28, 29]. 

Fitbit defines light-intensity PA as between 1.5 and 3 metabolic equivalents (METs), and moderate intensity PA as over 3 METs and below 6 METs (~ 50–69% of estimated maximum heart rate) [28, 30]. Vigorous-intensity PA is defined as activity ≥ 6 METs, or ≥ 145 steps per minute, corresponding to ~ 70–84% or ≥ 85% of max HR [29, 30]. We used the total daily minutes of each PA intensity and did not incorporate Fitbit’s Active Zone Minute weighing system in our analyses.

#### Demographic and disease-related factors

To describe the demographic characteristics of the study sample, we included age, ethnicity, race, employment, education level, and annual salary. In addition, to contextualize the identified phenotypes with respect to disease-relevant symptoms, we used the scores PROMIS questionnaires [14, 15] on pain and fatigue symptoms. Pain and fatigue are among the two most common symptoms reported by individuals with endometriosis [5]. The PROMIS pain and fatigue measures have been psychometrically evaluated in populations with chronic diseases [16]. Pain was assessed using a single item from the PROMIS v1.0 Pain Intensity 3a questionnaire (“In the past 7 days, how would you rate your pain on average?”) and the response options were numerical from a range of 0 (no pain) to 10 (worst pain imaginable) [14]. For fatigue assessment, *All of Us* includes a single item from the PROMIS Global Health (PROMIS-10) short form questionnaire (“In the past 7 days, how would you rate your fatigue on average?”) and the response options were categorical (i.e., “None”, “Mild”, “Moderate”, “Severe”, “Very Severe”, and “PMI: Skip”, indicating a skipped question within the *All of Us* workbench) [15]. 

### Statistical analysis

The functional data analysis (FDA) framework for our study can be summarized in 4 main steps: (1) data organization and cleaning, (2) smoothing our data into functional form or data curves over a selected time period, (3) model selection, and (4) characterization of PA patterns and associations with related symptoms.

#### Data organization and cleaning

For the analysis of habitual PA patterns, we selected 30 consecutive days to capture habitual PA over a sustained period, while balancing with data completeness. This trade-off allowed us to model daily PA trajectories and periodicities over a standardized period while minimizing missingness across participants. All the 30 consecutive days per participant were selected within the months of spring (March, April, and May) to reduce the potential influence of seasonality on PA volume. Each participant’s data were converted to wide format, and each participant’s row was stacked to create a 171 × 30 data matrix for functional clustering (*N* = 171).

The Fitbit data available in the *All of Us Researcher Workbench* do not include wear-time flags, making it difficult to distinguish between non-wear days and days of low activity. To preserve the integrity of the 30-day calendar window and avoid excluding potentially meaningful low-activity days (e.g., during symptom flares), we excluded days with step counts ≤ 500. This cut-off was selected based on previous studies using Fitbit devices [12, 31] and to minimize the risk of false positives (i.e., non-wear motion detected as PA by the device).

#### Missing data

The FMMs require all missing data to be imputed before model fitting. We imputed the days with missing daily step count data (up to 10 days per person within a 30-day span) using multiple imputation with chained equations via predictive mean matching (PMM) from the *MICE* package in R [32]. Multiple imputation is a well-established method for filling in missing data and has been applied in various fields and evaluated for its efficacy [32–34]. We generated 15 multiply-imputed datasets and used the fraction of missing information (FMI) to evaluate the quality of the imputations [35]. FMI provides a measure of the level of uncertainty of imputed data points in datasets with missingness [36] and is a common method for evaluating decisions involving multiple imputation [37, 38]. We conducted steps 2 and 3 (outlined below) on all imputed datasets and pooled the results, as per previously established guidelines [35, 36]. Full FMI results are provided in Supplemental Table S1.

#### Data smoothing (conversion to functional data)

The participants’ imputed data matrices were smoothed using a Fourier-transform. This process involves decomposition of raw data into different frequency components to represent the time series data as a sum of sine and cosine functions. This connects our data points of daily step counts and forms a curve of step count activity over a 30-day period. Fourier transforms are considered a suitable application to data that vary over time and potentially have periodic behavior [35] and have been demonstrated to perform well in previous similar studies [12].

#### Model specification and selection

For clustering, we implemented FMMs using the *funFEM library* in R. [39]. The *funFEM* package applies a discriminative FMM that clusters trajectories by projecting them into a lower-dimensional subspace designed to maximize between-cluster separation [39]. Within this subspace, a Gaussian mixture model is fit using a Fisher-EM algorithm, which iteratively estimates both the projection and cluster parameters until convergence [39]. Participants are assigned to clusters probabilistically via posterior probabilities, allowing classification uncertainty to be assessed [39]. The optimal number of clusters is selected using the Bayesian Information Criterion (BIC), which balances the model’s fit with its complexity, penalizing overly complex models to prevent overfitting [40]. Model selection was based on a combination of fit indices (Bayesian Information Criterion [BIC], Integrated Classification Likelihood [ICL]), posterior probabilities, and interpretability of the resulting clusters. Pooled model fit indices for all tested cluster resolutions using the Fourier basis and posterior probabilities (where applicable for converged models) are provided in Supplemental Table S2.

We tested FMMs with 2 to 6 cluster (K) resolutions using both Fourier and B-spline smoothing bases. The model assumes that the observed PA trajectories are generated from a mixture of underlying distributions, each representing a distinct cluster [39]. Accordingly, FMMs were fit to the functional data matrix to identify latent clusters of PA trajectories.

Cluster selection was informed by pooled model fit indices, including the Bayesian Information Criterion (BIC), Integrated Classification Likelihood (ICL), and Akaike Information Criterion (AIC), as well as cluster membership posterior probabilities. These metrics were used to balance model fit, parsimony, and interpretability. The raw (unsmoothed) daily step trajectories by cluster are provided in Supplemental Figure S1.

#### Characterization of model phenotypes

After selecting the best-fitting model, we characterized the identified phenotypes based on the 30-day averages and their variabilities (SD) for all PA parameters. We used linear mixed regression models for pairwise comparisons where we regressed each of the PA parameters on the phenotypes, as well as the participant as a random intercept. Regression models were implemented using the *lmerTest* package in R [41].

Next, we compared PROMIS-based pain and fatigue scores across phenotypes for a subset of 129 participants who completed these surveys after their PA data window for a more disease-specific contextualization. We calculated the frequencies of responses for PROMIS pain and fatigue across phenotypes for our analysis. We compared differences in pain and fatigue scores using the Kruskal-Wallis test for non-normal data and Chi-square test of independence, respectively.

## Results

### Sample characteristics

Out of 825 eligible individuals with a diagnosis of endometriosis, 291 had Fitbit data available. The final analytic sample based on the allowable missingness in the given 30-day period included 171 participants, yielding 4,795 person-level days of data in total for analysis. The demographics of the study sample are provided in Table 1. The mean age was 57.7 years (SD = 14.3 years, Range = 24–91 years). Briefly, *n* = 153 (89.5%) self-identified as Not Hispanic or Latino, with *n* = 142 (83%) self-identifying as White. Demographics were reported in this manner to comply with the All of Us Data and Statistics Dissemination Policy [42].

**Table 1:** *All of Us* study sample demographics

All of us study sample demographics	*N* = 171
Mean Age in years (SD)	57.7 (14.3)
Ethnicity	**N (%)**
Not Hispanic or Latino	153 (89.5%)
Hispanic or Latino	< 20 (< 11.7%)
Unknown	< 20 (< 11.7%)
Race	
White	142 (83.0%)
Black	< 20 (< 11.7%)
Unknown	< 20 (< 11.7%)
Asian	< 20 (< 11.7%)
Mixed Race	< 20 (< 11.7%)
Employment	
Employed	< 87 (< 51.0%)
Unemployed	77 (45.0%)
Unknown	< 20 (< 11.7%)
Education Level	
College or Advanced Degree	116 (67.8%)
Highest Grade: Some College	32 (18.5%)
Highest Grade: 12 or GED	< 20 (< 11.7%)
Less than High School or Equivalent	< 20 (< 11.7%)
Unknown	< 20 (< 11.7%)
Annual Income	
Less than $25,000	< 20 (< 11.7%)
$25,000 - $50,000	26 (15.0%)
$50,000 - $75,000	29 (16.8%)
$75,000 - $100,000	20 (11.6%)
$100,000 - $150,000	34 (19.7%)
More than $150,000	< 33 (< 19.3%)
Unknown	21 (12.3%)

Overview of cohort demographics including mean age (SD), ethnicity, race, employment, education level, and annual income (N (%)). “<20” is used to remain in compliance with the *All of Us* Research Program Data Dissemination Policy, which states that participant level N’s of < 20 cannot be reported in tables or figures

### Model results

**Fig. 1:**
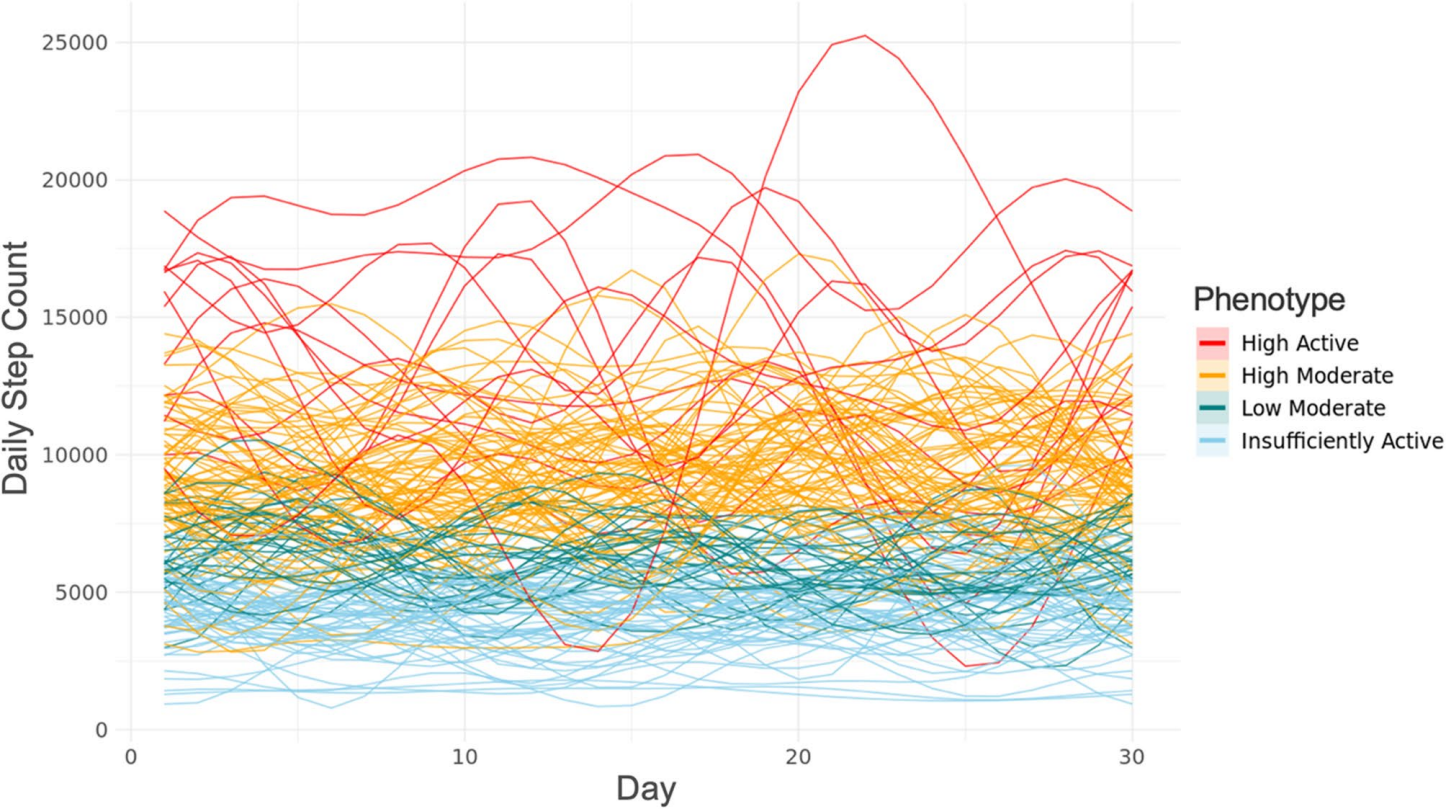
Smoothed curves of daily step count data by phenotype. Fourier basis functions of functional mixture model (FMM)-identified phenotypes representing the daily step count activity of each participant (*N* = 171) smoothed over 30 days. Red curves indicate the “High Active” phenotype, orange curves indicate the “High Moderate” phenotype, green curves indicate the “Low Moderate” phenotype, and light blue curves indicate the “Insufficiently Active” phenotype

**Fig. 2:**
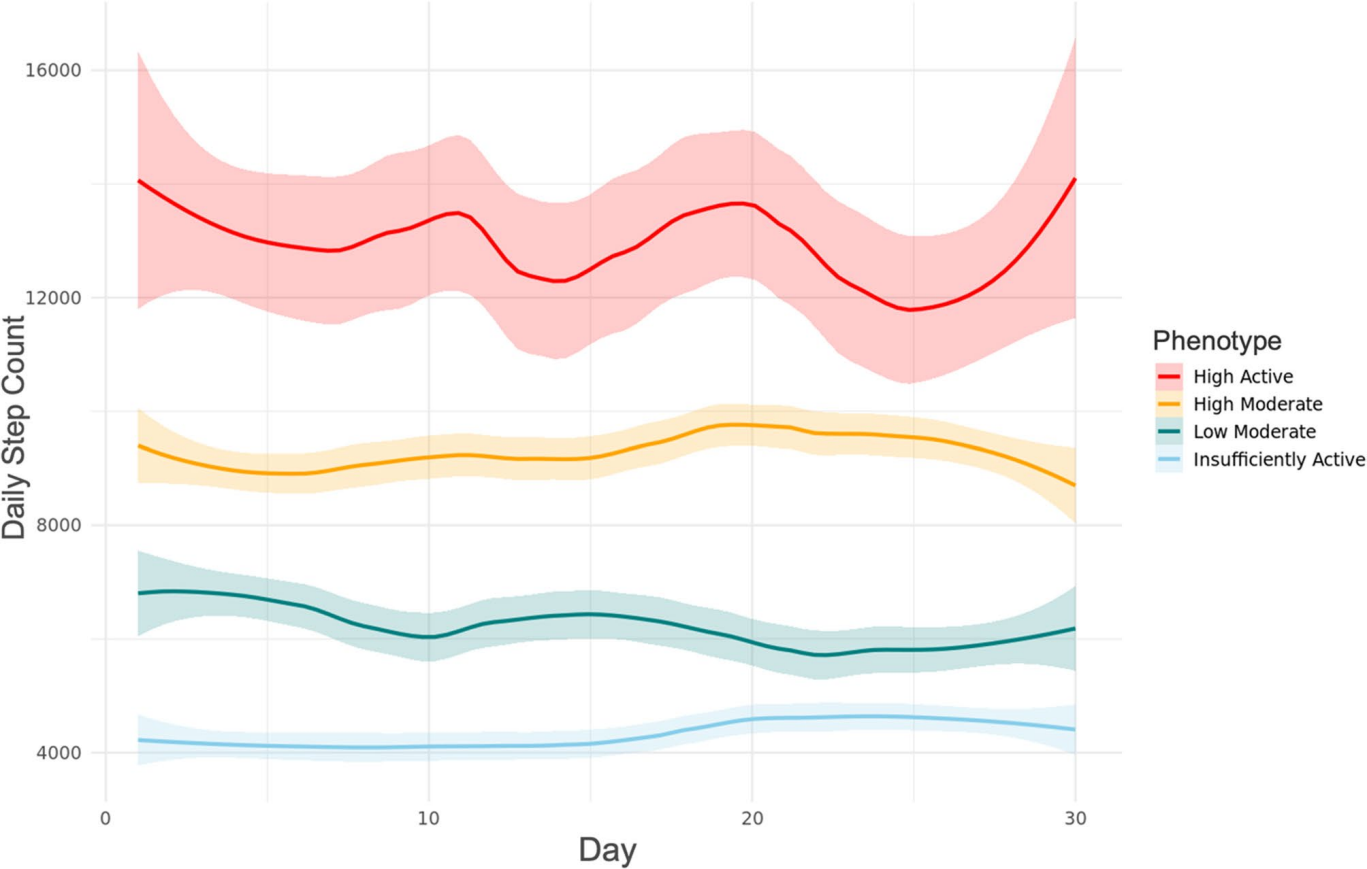
Average smoothed curves of daily step counts by phenotype. Mean of the smoothed curves of step counts for the functional mixture model (FMM)-identified phenotypes over the 30-day period. Red curve indicates the “High Active” phenotype, orange curve indicates the “High Moderate” phenotype, green curve indicates the “Low Moderate” phenotype, and light blue curve indicates the “Insufficiently Active” phenotype. Colored bounds around smoothed curves indicate 95% confidence intervals calculated using the pooled estimates from the imputed datasets, and represent the standard error around the smoothed mean trajectory for each phenotype

The final best-fitting model indicated a 4-cluster solution (K = 4) using a Fourier basis, based on the combination of fit indices (BIC = −11,710, AIC = −11,638, ICL = −11,675), interpretability of the phenotypes, and clearer separation in PA patterns. These clusters represented distinct PA trajectories among women with endometriosis, varying in both volume and variability of activity over time. We refer to these phenotypes as “Insufficiently Active,” “Low Moderate,” “High Moderate,” and “High Active.” Figs. 1 and 2 respectively display the person-level and phenotype-level Fourier-smoothed step count trajectories over 30 days. Supplemental Figure S2 shows the distribution of participants across the 4-cluster solution in the discriminative space of the FMM.

Model fit results for all tested cluster resolutions using the Fourier basis are provided in Supplemental Table S2, and corresponding B-spline smoothing results are shown in Supplemental Table S3 and Figure S3. The median membership posterior probability was 0.93 (SD = 0.048, range = 0.79–0.98), indicating high degree of model confidence in assigning participants to their respective clusters (also shown in Supplemental Table S3).

### Phenotype characterization

Phenotype-level descriptive statistics on all PA parameters are provided in Table 2.

**Table 2:** Phenotype-level daily step counts and physical activity (PA) means and ranges

PA Variable	Day-Level Mean (SD)	Range
Step Counts		
Insufficiently Active	4317.1 (2110.8)	504–17,693
Low Moderate	6234.0 (2515.8)	752–16,169
High Moderate	9283.9 (3661.2)	534–26,486
High Active	12918.8 (5606.4)	540–29,368
Lightly Active Minutes		
Insufficiently Active	148.4 (84.1)	0–577
Low Moderate	201.2 (97.3)	0–726
High Moderate	230.0 (132.2)	0–1440
High Active	265.7 (147.4)	0–796
Fairly Active Minutes		
Insufficiently Active	12.1 (23.4)	0–242
Low Moderate	10.1 (19.5)	0–144
High Moderate	39.6 (49.5)	0–335
High Active	43.8 (46.9)	0–234
Very Active Minutes		
Insufficiently Active	5.1 (10.9)	0–138
Low Moderate	8.5 (17.7)	0–164
High Moderate	18.6 (32.3)	0–480
High Active	31.5 (36.2)	0–182
MVPA Minutes		
Insufficiently Active	17.2 (28.9)	0–253
Low Moderate	18.6 (32.3)	0–247
High Moderate	58.2 (59.6)	0–480
High Active	75.2 (64.6)	0–300

Phenotype-level daily step count and PA minutes means and ranges (“Insufficiently Active”: *n* = 57, “Low Moderate”: *n* = 27, “High Moderate”: *n* = 74, “High Active”: *n* = 13)

The “High Active” phenotype was the smallest (*n* = 13) and was associated with the highest daily mean steps and PA minutes for all intensities. Of note, the *“*High Active” phenotype was also associated with the highest day-to-day variance in steps. The *“*High Moderate*”* phenotype was the largest (*n* = 74) and had fewer daily steps compared to “High Active” but similar minutes of MVPA (i.e., 58.2 vs 75.2 min, respectively, *p* = 0.117 – see Table 3). The “Low Moderate” phenotype (*n* = 27) had lower daily steps and minutes of PA in comparison to “High Moderate”, as well as lower variability in these PA metrics. The “Low Moderate” phenotype overall had slightly higher volumes for all PA intensities, except for fairly active minutes than the *“*Insufficiently Active*”* phenotype (*n* = 57), along with higher variation (see Table 2). The “Insufficiently Active” phenotype was the second largest (*n* = 57) and was characterized by the fewest number of daily steps and minutes of PA intensities and lowest day-to-day variation, except for fairly active minutes. We provide boxplots depicting the distributions of day-level step counts and all intensities of PA for these phenotypes in Supplemental Figure S4A-E.

**Table 3:** Linear mixed regression model results comparing step counts, lightly active and mvpa minutes between phenotypes

Comparison	Estimate	Std. Error	t-value	*p*-value
Step Counts				
High Moderate (Intercept)	9280.8	153.5	60.466	< 0.001*
High Moderate – Insufficiently Active	−4989.2	232.7	−21.442	< 0.001*
High Moderate – High Active	3685.4	397.1	9.282	< 0.001*
High Moderate – Low Moderate	−3064.4	296.9	−10.323	< 0.001*
Lightly Active Minutes				
High Moderate (Intercept)	230.035	9.114	25.239	< 0.001*
High Moderate – Insufficiently Active	−81.554	13.817	−5.902	< 0.001*
High Moderate – High Active	35.621	23.578	1.511	0.133
High Moderate – Low Moderate	−28.83	17.628	−1.635	0.104
MVPA Minutes				
High Moderate (Intercept)	58.155	4.189	13.882	< 0.001*
High Moderate – Insufficiently Active	−41.039	6.351	−6.462	< 0.001*
High Moderate – High Active	17.091	10.837	1.577	0.117
High Moderate – Low Moderate	−39.587	8.102	−4.886	< 0.001*

Results of linear mixed regression models comparing mean daily step count, mean daily lightly active minutes, and mean daily moderate-to-vigorous (MVPA) across phenotypes. Comparisons are between the “High Moderate” (intercept) and “High Active” phenotypes, “High Moderate” and “Low Moderate” phenotypes, and “High Moderate” and “Insufficiently Active” phenotypes

**Fig. 3:**
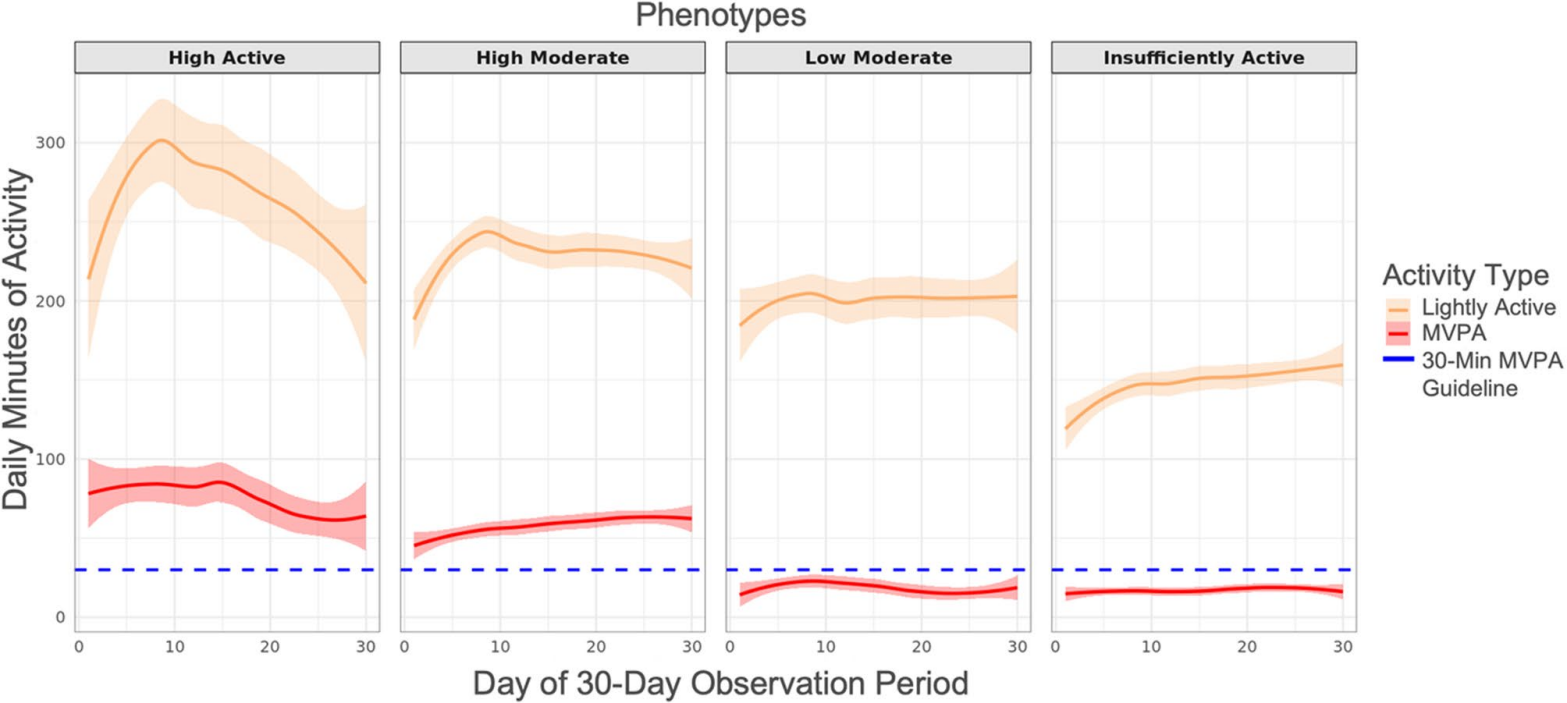
Daily Lightly Active and MVPA Patterns by Phenotype. Phenotype-level means of daily lightly active minutes and moderate-to-vigorous (MVPA) minutes over 30 days. Red curves indicate MVPA minute trajectories. Orange curves indicate lightly active minute trajectories. Colored areas surrounding curves indicate 95% confidence intervals around lightly active and MVPA minutes over the 30-day period. Blue dashed line indicates the U.S. Department of Health and Human Services (USDHHS) recommended minimum daily MVPA amount of 30 min

Comparisons of daily lightly active and MVPA minutes are shown in Fig. 3 where the overall trend of reduced volume and variability is visible as the phenotypes move from most active (“High Active”) to least active (“Insufficiently Active”). The blue dashed line in Fig. 3 represents the recommendation of 30 min per day of MVPA per the U.S. Department of Health and Human Services (USDHHS) on PA for adults [43].

### Characterization of model-identified phenotypes

Results from the linear regression models comparing daily PA parameters across phenotypes are provided in Table 3.

All point estimates for step counts were statistically significant (*p* < 0.001; see Table 3). For lightly active minutes, the “High Moderate” phenotype was associated with significantly higher minutes than the “Insufficiently Active” phenotype (*p* < 0.001), but non-significant differences in minutes were observed compared to the “High Active” and “Low Moderate” phenotypes (*p* = 0.133 and *p* = 0.104, respectively). For MVPA minutes, the “High Moderate” phenotype significantly differed from the “Low Moderate” and “Insufficiently Active” phenotypes (*p* < 0.001) and was associated with significantly higher minutes than both phenotypes (*p* < 0.001 for both). However, the difference in MVPA between *“*High Active*”* and *“*High Moderate*”* was not statistically discernable (*p* = 0.117).

### Comparisons of reported pain and fatigue levels by phenotype

**Fig. 4:**
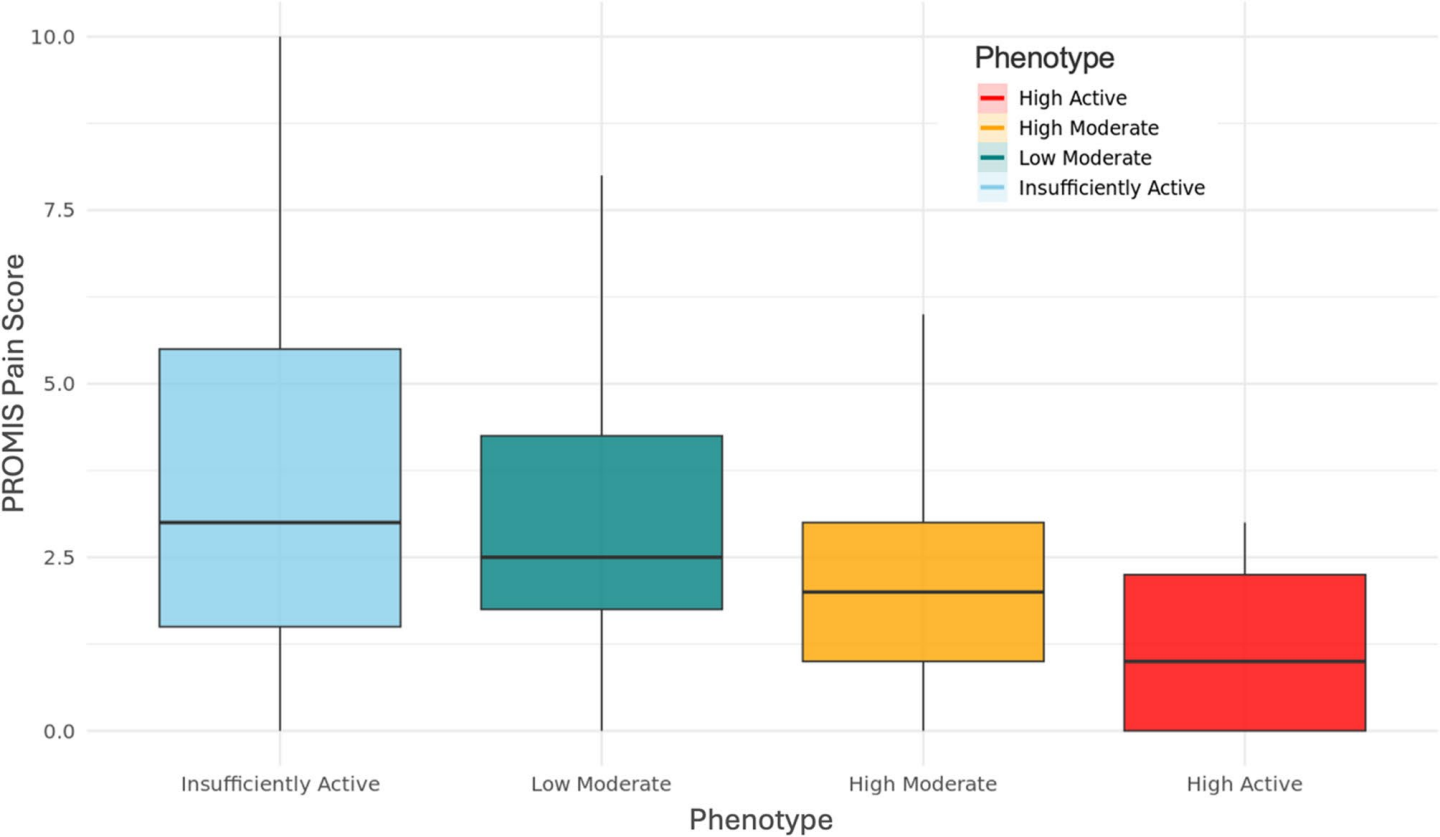
PROMIS pain score distribution by phenotype. Scores range from 0 (no pain) to 10 (worst pain imaginable). Boxplots display the median (solid black line), interquartile range (box), and whiskers extending to 1.5× IQR. Colors correspond to phenotype labels

**Fig. 5:**
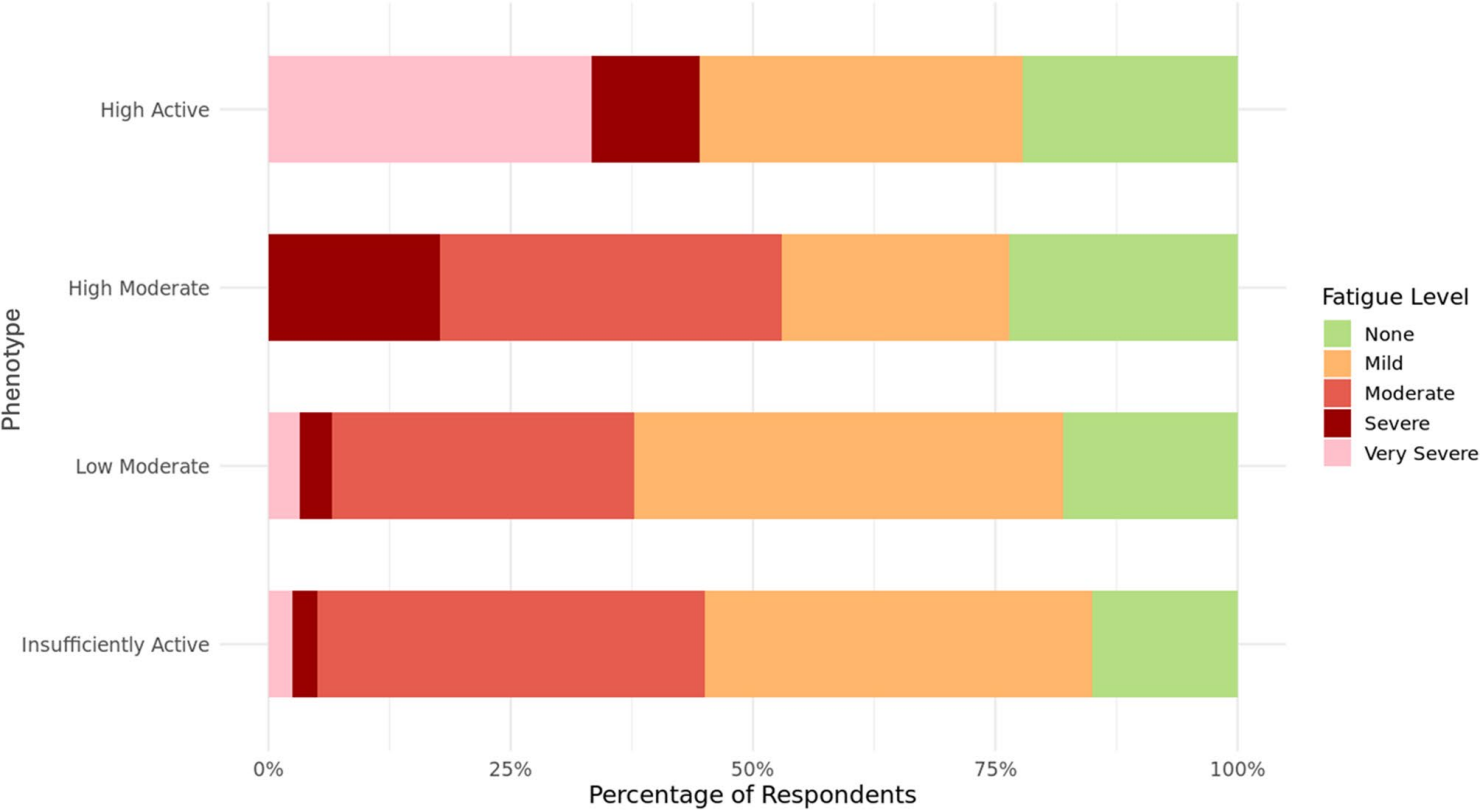
Proportion of PROMIS fatigue responses by phenotype. PROMIS fatigue responses by phenotype as a proportion of the total number of participants within each phenotype

Figures 4 and 5 depict the phenotype-level scores from the PROMIS pain and fatigue questionnaires, respectively. Figure 4 shows PROMIS pain scores by phenotype using boxplots. *Mean* PROMIS pain scores are as follows: “High Active” (1.88), “High Moderate” (2.34), “Low Moderate” (3.13), and “Insufficiently Active” (3.59). Figure 5 presents the proportions of self-reported fatigue severity levels by phenotype. The “Low Moderate” and “Insufficiently Active” phenotypes had the highest proportions of “Mild” fatigue (44% and 40%, respectively), while the “High Moderate” phenotype had the highest proportion of “Moderate” fatigue (35%). Notably, the “High Active” phenotype reported the highest proportion of “Very Severe” fatigue (33%), and the “Insufficiently Active” group had the lowest proportion of “None” fatigue responses (15%).

There were significant differences in PROMIS pain scores across phenotypes based on the Kruskal-Wallis test (χ²(3) = 271.8, *p* < 0.001). Follow-up pairwise Wilcoxon rank-sum tests indicated significant differences between all phenotype pairs (adjusted *p* < 0.001). PROMIS fatigue severity levels also differed significantly across clusters (Pearson’s χ²(12) = 875.36, *p* < 0.001). All statistical test results for PROMIS pain and fatigue are provided in Supplemental Table S4.

## Discussion

In this study, we investigated PA trajectories over a 30-day period in a sample of women with endometriosis. We used the FDA framework to investigate PA trajectory clusters, which leverages data curves instead of aggregated data points. This retains more information (e.g. temporal trends) than otherwise, enabling a more comprehensive analysis of PA patterns. Our results indicated four distinct phenotypes characterized by differences in PA volume and variability. In exploratory analyses, self-reported pain and fatigue levels also varied among phenotypes, pointing to possible beneficial effects of PA on pain. Our findings highlight the potential of analyzing PA trajectories using an FDA framework, especially in a population impacted by chronic pain.

The results from the best-fitting model indicated 4 distinct phenotypes of PA patterns based on daily step counts, further characterized by differences in total daily minutes and inter-day variances in the PA parameters. The *“*High Active*”* phenotype was characterized by the highest volume and variability of PA over the 30-day observation periods. The *“*High Active*”* phenotype had the highest average MVPA minutes among all phenotypes with 75.2 min/day. The *“*High Moderate*”* phenotype was characterized by a relatively lower average step count and MVPA to the “High Active” phenotype (see Table 2). The *“*Low Moderate*”* phenotype was characterized by a lower average step count than the “High Moderate” phenotype, but a substantial decrease in average MVPA minutes over time compared to the “High Active” phenotype. The “Insufficiently Active” phenotype was characterized by the lowest average step count (4317.1 steps/day) and lowest average MVPA minutes. The “Insufficiently Active” phenotype also had the lowest variance for step count over the 30-day observation periods (SD = 2110.8). Future studies should incorporate longitudinal PA data to examine cluster stability over time and whether individuals maintain the same phenotype across different observation periods.

Our step count findings are consistent with previous research on special populations (such as older individuals and those with chronic illnesses), which report daily step counts ranging from 1,200 to 8,800 steps [44]. Our analyses suggest that the “Insufficiently Active” and *“*Low Moderate*”* phenotypes are more likely to fall within this range. Although the study on special populations did not specifically include women with endometriosis, our results suggest that some individuals in our sample surpass the expected PA levels for those with other chronic illnesses (i.e. diabetes, breast cancer, fibromyalgia, arthritis, neuromuscular diseases) [44]. 

In the *“*High Active*”* phenotype, the average daily MVPA minutes (i.e., 75.2 min) was more than twice the daily recommended amount (30 min/day) by the USDHHS [43]. The *“*High Moderate*”* phenotype was similarly associated with 58.2 min/day of MVPA. These findings suggest that individuals in the *“*High Active*”* and *“*High Moderate*”* phenotypes are most likely to accumulate at least the minimum amount of PA recommended for overall health on most days. In contrast, the *“*Low Moderate*”* and “Insufficiently Active” phenotypes were associated with significantly lower daily MVPA min (i.e., 18.6 and 17.2 min/day, respectively). Both the “Low Moderate” and “Insufficiently Active” phenotypes do not meet the weekly threshold of 150 min/week set by the USDHHS [43]. The PA patterns of these phenotypes also indicated lower variability in PA over time, suggesting these individuals tend to remain consistent within their respective lower activity patterns.

Our analyses further indicated that PROMIS pain scores were inversely related to PA volume, where the *“*High Active*”* phenotype was associated with the lowest pain levels based on the PROMIS pain questionnaire. A cross-sectional cohort study comparing PROMIS 7-day pain intensity between controls and chronic conditions (osteoarthritis, premenstrual syndrome, hernia repair, and breast cancer) demonstrated that mean pain intensity for the control group was ~ 2 on the 0–10 scale [45]. In comparison, the individuals in our *“*High Active*”* phenotype have slightly lower average pain scores than controls from this study (i.e., 1.88), the *“*High Moderate*”* phenotype has slightly higher average pain scores (i.e. 2.34), and the average pain scores for the *“*Low Moderate*”* and “Insufficiently Active” phenotypes are even higher (i.e. 3.13 and 3.59, respectively). This provides further support to the notion that increased habitual PA may play a role in mitigating pain associated with endometriosis.

Given the cross-sectional nature of our data, we cannot infer causality. It is possible that lower pain levels enabled greater PA, rather than PA leading to reduced pain. To address this within the limits of the available data, we used PROMIS pain and fatigue scores that were collected after the 30-day PA windows used in the analyses. While this temporal ordering strengthens the plausibility of interpreting PA as a possible antecedent of symptom levels, it does not eliminate the potential for reverse causality. As such, the bidirectional relationship between PA and symptoms warrants further investigation through prospective or longitudinal designs.

Our findings are further in line with a review supporting the underlying physiological mechanisms for PA related analgesia, which lends biological plausibility to our results [46]. This is noteworthy given that previous research indicates a strong association between regular MVPA and improved symptom management and quality of life in individuals with chronic conditions, including endometriosis [5, 7, 31, 47, 48]. To our knowledge, this is the first study reporting this association in endometriosis based on trends of PA using objectively estimated data.

In contrast to pain scores, PROMIS fatigue severity levels also differed significantly across clusters (see Supplemental Table S4), supporting that each phenotype represents a distinct fatigue severity distribution. While the “Low Moderate” phenotype had the highest proportion of “Mild” fatigue responses (44%), the “High Active” phenotype reported the highest proportion of “Very Severe” fatigue (33%). The “High Moderate” phenotype showed the greatest combined proportion of “Moderate” and “Severe” fatigue responses (53%), while the “Insufficiently Active” phenotype had the lowest proportion of “None” responses (15%) and a high proportion of “Moderate” fatigue (40%).

In summary, these findings suggest that both insufficient and high levels of PA may be associated with elevated fatigue burden, though the nature of this burden differs by phenotype. Fatigue can be difficult to measure and capture [49], therefore more research needs to be done to confirm this interaction. These results may reflect the complex and potentially nonlinear relationship between PA and fatigue in individuals with endometriosis, where higher activity may coincide with increased symptom burden in some cases. Alternatively, this pattern may reflect that high levels of PA can co-occur with high fatigue severity in some individuals, potentially due to unmeasured contextual or behavioral factors. However, without more contextual data such as daily routines, occupational activity, or coping strategies, this interpretation remains preliminary. Future research, particularly qualitative and longitudinal, can explore individual variability and motivations to better elucidate these patterns.

Although research on clustering PA behavior among chronic disease populations is limited, our study shares some similarities with those from another clustering study [50], who also identified four PA patterns using accelerometry-based step counts in participants with high cholesterol, hypertension, arthritis, and other circulatory conditions: “inactive- sedentary,” “low activity,” “active,” and “very active.” Their average step counts were 4,328, 8,158, 12,681, and 17,982 steps/day, respectively, with MVPA averages of 7.7, 25.5, 52.2, and 94.4 min/day. Our findings, which also identified four clusters, indicate that the “Insufficiently Active” phenotype averaged fewer than 5,000 steps/day, with the two lowest activity phenotypes not meeting the 30 min/day USDHHS recommendation for MVPA. In line with this clustering study [50] which reported that lower PA levels were associated with higher morbidity and healthcare utilization, our results support the notion that women with lower PA levels are more likely to face greater health challenges, consistent with previous literature on chronic diseases.

These results collectively point to the complexity of the relationship between PA and endometriosis symptoms, such as pain and fatigue. While the *“*High Active*”* phenotype had the greatest levels of PA, they recorded a substantial proportion of “Very Severe” fatigue even though their pain levels were the lowest among all phenotypes. This varied relationship between PA and pain, and PA and fatigue potentially highlights that increased PA can reduce pain but does not necessarily decrease fatigue levels in this population. Alternatively, optimal ranges of PA could vary based on individual needs to maximize its benefits for endometriosis symptom management (i.e., reduce or prevent pain without exacerbating fatigue). It is possible that for some individuals, a consistent distribution of PA, as opposed to intense bouts of PA, is more efficacious for flare-up prevention [6]. Similarly, regularity might be more important than the intensity in this context [5]. Future studies should further investigate this relationship in larger samples, with additional disease-specific and temporally aligned self-report data to further contextualize PA behavior.

Finally, the observed age distribution (spanning several decades; see Supplemental Table S5) presents a unique opportunity to study PA maintenance across different life stages among those with endometriosis. Given the paucity of research across the lifespan among those with endometriosis as well as the delay in endometriosis diagnosis, the age diversity in this study aligns with the *All of Us* cohort’s intentional inclusion of underrepresented older populations in chronic disease research. It also strengthens generalizability to clinical populations while highlighting the need for age-specific PA recommendations in chronic gynecological conditions. Nevertheless, more focused future studies are warranted to stratify analyses by menopausal status, as estrogen fluctuations may differentially impact both endometriosis symptoms and PA patterns.

### Limitations

This study has several limitations. First, we use retrospective, secondary data from the *All of Us Research Program*, which uses self-report surveys that do not include some of the variables that could potentially be relevant to our analysis, such as habitual PA behaviors, other health behaviors, menstrual cycle status or health, or pain medication use at the time that corresponds to the Fitbit-based PA data. As such, our ability to contextualize the phenotypes was limited to the available variables (e.g., pain, fatigue) for assessing symptom fluctuations and their relationship to PA. Furthermore, because these PROMIS symptom surveys were administered independently of the PA collection window, their timing relative to the 30-day PA period varied substantially across participants.

Additionally, we used 30 consecutive days of data limited to spring months (March, April, May) to reduce potential seasonal influence and introduce some level of standardization across individuals. This was also based on data availability, i.e., these months had more data available and less intermittent missingness overall compared to other months. However, we acknowledge that this hinders us from examining seasonal variations in PA patterns that could be relevant. Third, 83% of participants identified as White and 89.5% as non-Hispanic, restricting the generalizability of findings to more diverse populations.

Similarly, our analysis is limited to individuals who own Fitbit devices and consented to data sharing, introducing potential bias where the sample may represent individuals who are already more active. This would suggest a possible overestimation of PA levels in our sample, which may limit the applicability of findings to more inactive individuals or those less likely to use wearables for PA tracking. Additionally, Fitbit’s algorithms used to estimate PA intensity could have included misclassification error, especially in free-living settings or among individuals with variable gait patterns [51]. 

The High Active phenotype had the lowest average age, which may partially explain their ability to sustain higher levels of PA. Nevertheless, the observed clustering patterns remain valuable for highlighting meaningful variability in activity and symptom experiences across individuals with endometriosis. Accordingly, future studies can focus on prospective PA data collection striving to enroll participants from diverse backgrounds. This would enable more comprehensive identification of those who stand to benefit the most from targeted, personalized PA for symptom management, especially in understudied populations (minority races/ethnicities and sexual and gender minorities).

## Conclusions

This study demonstrates the utility of FDA in characterizing PA trajectories among women with endometriosis, providing insight into the complex relationship between habitual PA volume, intensity, and symptom management in a chronic pelvic pain population. The results from the FMMs based on step counts revealed four distinct phenotypes of PA patterns. These phenotypes displayed different magnitudes of variance over the 30-day period in PA parameters, in addition to total volume. Exploratory analyses also suggested an inverse association between PA volume and self-reported pain, raising the possibility that greater habitual activity may be linked to lower disease-related pain. However, the cross-sectional nature of our data and the variability in symptom survey timing limit causal inference and warrant further longitudinal studies.

Phenotyping enables identification of those at greater risk for physical inactivity and who could benefit most from personalized intervention approaches to improve PA behavior. A nuanced approach to personalized PA recommendations may support quality of life in this population, but more studies are needed to confirm these associations. These findings collectively underscore the potential benefits of integrating personalized PA interventions into symptom management plans, particularly for women aiming to leverage PA as part of their endometriosis management plan.

## Supplementary Information

Supplementary Material 1

## Data Availability

Data used for this manuscript are obtained from the *All of Us Research Program* and not owned by the authors. Data from the *All of Us Research Program* are accessible only through the Researcher Workbench (https://workbench.researchallofus.org), as stipulated in the informed consent of participants in the program. This data use agreement prohibits investigators from providing row level data on All of Us participants and thus providing a de-identified dataset is not possible for this manuscript. The *All of Us Data and Statistics Dissemination* Policy also stipulates that “No data or statistics can be reported that allow a participant count of 1 to 20 to be derived from other reported cells or information, including in text, tables, or figures. This includes the use of percentages or other mathematical formulas that in combination would allow an individual to deduce a participant count of less than 20”, requiring participant-level counts less than 20 to be reported as <20 in tables and figures. The authors received an exception to the All of Us Data and Statistics Dissemination Policy from the Resource Access Board. This study was completed in compliance with all the policies outlined in the *Overview of All of Us Research Program Policies for Researchers*.* The code used for this demonstration project is available within the Researcher Workbench at https://workbench.researchallofus.org/workspaces/aou-rw-bd78ba22/dupv6datafemalereproductivedisordersacrossdiversepatientpopulations/data. Any investigator interested in accessing data used for this manuscript or any other All of Us Research Program data can do so following the procedures outlined in https://www.researchallofus.org/apply/.

## Abbreviations

PA: Physical activity
FMM: Functional mixture models
BIC: Bayesian Information Criterion
PROMIS: Patient-Reported Outcomes Measurement Information System
MVPA: Moderate-to-vigorous physical activity
QoL: Quality of life
CDR: Curated Data Repository
EHR: Electronic Health Record
MET: Metabolic equivalents
FDA: Functional data analysis
MICE: Multivariate Imputation by Chained Equations
PMM: Predictive mean matching
FMI: Fraction of missing information
USDHHS: U.S. Department of Health and Human Services
