# Supplementary material for: Physical activity phenotypes in endometriosis using unsupervised learning via functional mixture models
Source: BMC Womens Health. 2025 Dec 11;26:29. doi: 10.1186/s12905-025-04087-2 (PMC12802236; doi:10.1186/s12905-025-04087-2)
Supplement: Supplementary file 1 — Supplementary Material 1 [file 12905_2025_4087_MOESM1_ESM.docx]

**Supplemental Materials**

**Supplemental Table S1: Fraction of Missing Information after Multiple Imputation of Daily Step Count Dataset**

| **Variable** | **FMI** |
| --- | --- |
| Day 1 | 0.0006 |
| Day 2 | 0.0006 |
| Day 3 | 0.0007 |
| Day 4 | 0.0002 |
| Day 5 | 0.0004 |
| Day 6 | 0.0004 |
| Day 7 | 0.0001 |
| Day 8 | 0.0001 |
| Day 9 | 0.0001 |
| Day 10 | 0.0001 |
| Day 11 | 0.00 |
| Day 12 | 0.00 |
| Day 13 | 0.0001 |
| Day 14 | 0.00 |
| Day 15 | 0.0001 |
| Day 16 | 0.0001 |
| Day 17 | 0.00 |
| Day 18 | 0.00 |
| Day 19 | 0.00 |
| Day 20 | 0.0001 |
| Day 21 | 0.00 |
| Day 22 | 0.0002 |
| Day 23 | 0.0002 |
| Day 24 | 0.0005 |
| Day 25 | 0.0004 |
| Day 26 | 0.0004 |
| Day 27 | 0.0004 |
| Day 28 | 0.0007 |
| Day 29 | 0.0006 |
| Day 30 | 0.0005 |
| **Mean FMI** | 0.0003 |

**Supplemental Table S1:** Fraction of missing information (FMI) by imputed days after multiple imputation (MI) via predictive mean matching (PMM). Mean FMI shown at the end of the table.

**Supplemental Table S2: Pooled model fit indices and posterior probabilities for functional mixture models using Fourier smoothing**

| **K** | **Mean BIC** | **Mean AIC** | **Mean ICL** | **Posterior Mean (SD)** | **Posterior Median (MAD)** |
| --- | --- | --- | --- | --- | --- |
| 2 | -11744.52 | -11724.1 | -11733.44 | NA | NA |
| 3 | -11718 | -11674 | -11698 | 0.956 (0.019) | 0.995 (0.0073) |
| 4 | -11710 | -11638 | -11675 | 0.916 (0.062) | 0.930 (0.048) |
| 5 | -11740.91 | -11635.67 | -11695.7 | NA | NA |
| 6 | -11779.61 | -11636.67 | -11732.5 | NA | NA |

**Supplemental Table S2:** Pooled model fit indices—Bayesian Information Criterion (BIC), Akaike Information Criterion (AIC), and Integrated Completed Likelihood (ICL)—for each tested cluster resolution (K=2:6) using *Fourier* smoothing. Posterior probabilities (mean and median with corresponding standard deviation [SD] or median absolute deviation [MAD]) are reported for cluster resolutions where models successfully converged and were selected as optimal in at least one imputed dataset (N=171). NA indicates that posterior probabilities could not be obtained due to model instability or lack of selection across imputations. Converged models included K=3 and K=4, for which the posterior probabilities are shown.

**Supplemental Figure S1: Raw 30-Day Step Trajectories by Cluster**

**
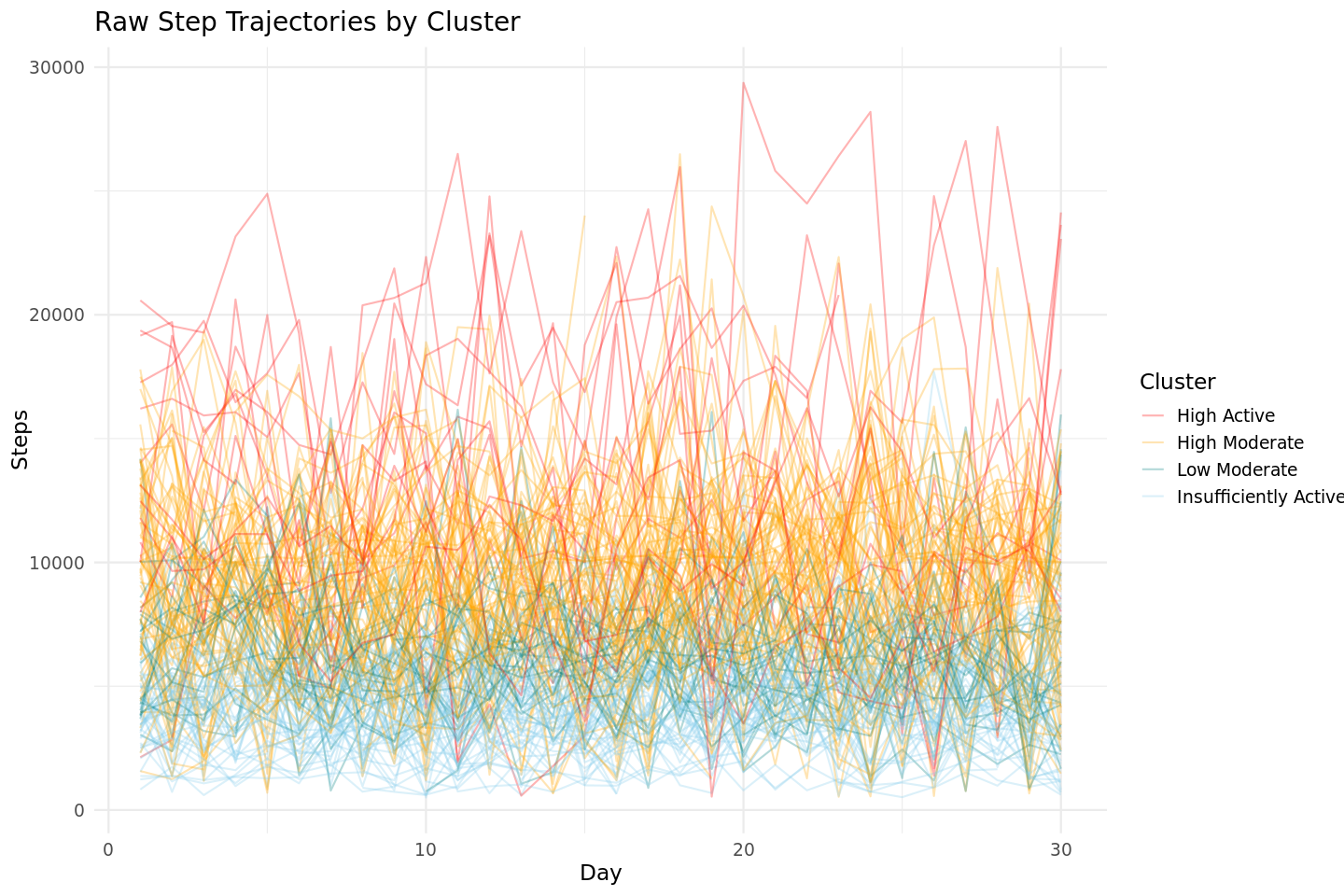
**

**Supplemental Figure S1:** Unsmoothed daily step trajectories over 30 consecutive days for all participants (N=171), colored by final cluster membership derived from “funFEM” clustering with label switching (K = 4). The high degree of day-to-day variability highlights the importance of functional data analysis and smoothing to reveal meaningful behavioral patterns across clusters.

**Supplemental Figure S2: Functional Mixture Model (FMM) Coefficients in Discriminative Space Within Model**


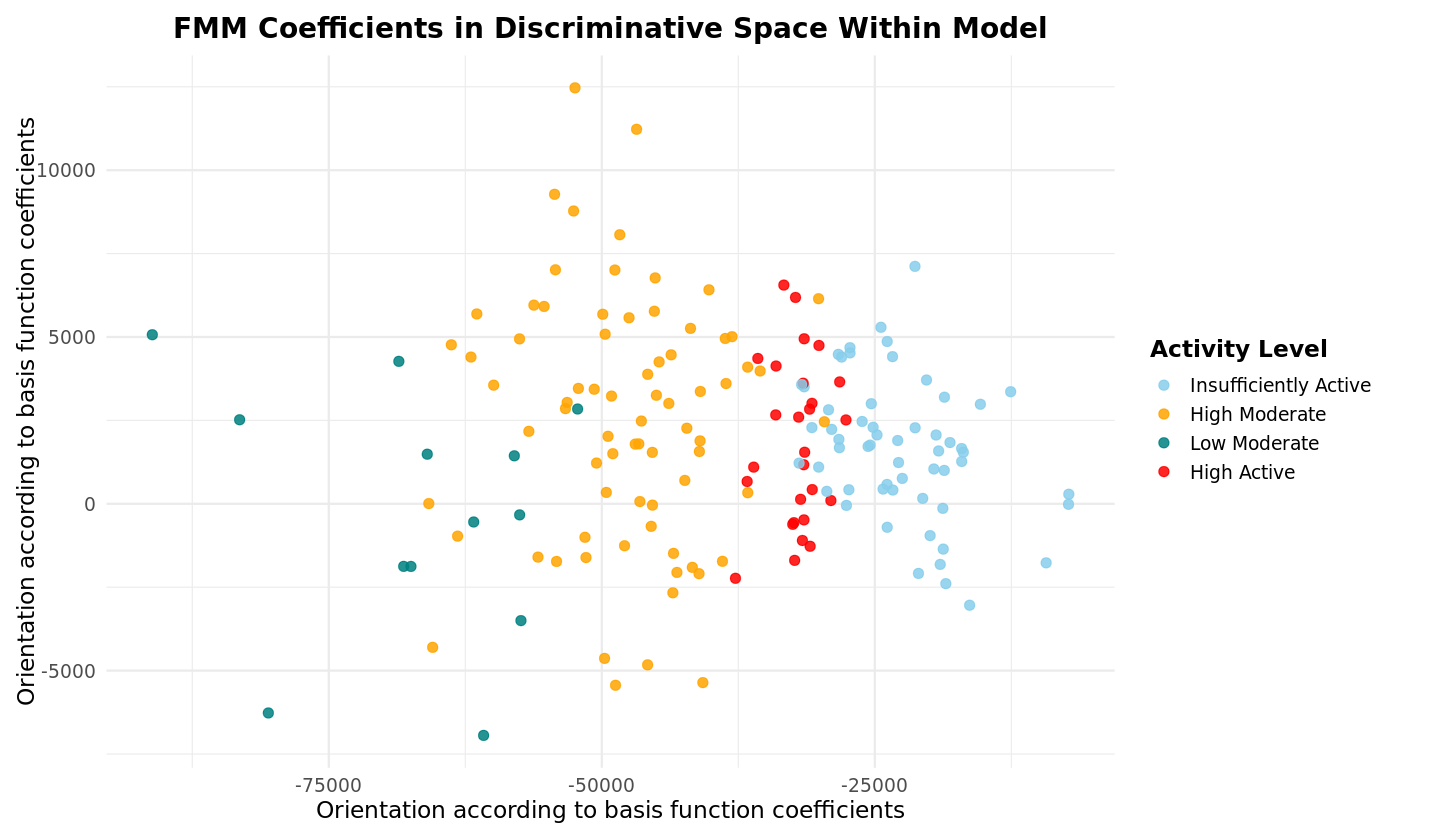


**Supplemental Figure S2:** Individual functional mixture model (FMM) coefficients within the discriminative space of the clustering model. Each dot represents 1 participant (N=171). Red dots indicate the “High Active” cluster, orange dots indicate the “High Moderate” cluster, green dots indicate the “Low Moderate” cluster, and light blue dots indicate the “Insufficiently Active” cluster.

**Supplemental Table S3: Pooled model fit indices and posterior probabilities for functional mixture models using B-spline smoothing**

| **K** | **Mean BIC** | **Mean AIC** | **Mean ICL** | **Posterior Mean (SD)** | **Posterior Median (MAD)** |
| --- | --- | --- | --- | --- | --- |
| 2 | -16814.97 | -16789.84 | -16801.66 | 0.967(0.091) | 0.999(0.00019) |
| 3 | -17114.08 | -17060.68 | -17094.99 | 0.905(0.14) | 0.983(0.025) |
| 4 | -17266.32 | -17179.93 | -17254.1 | NA | NA |
| 5 | NA | NA | NA | NA | NA |
| 6 | NA | NA | NA | NA | NA |

**Supplemental Table S3:** Pooled model fit indices—Bayesian Information Criterion (BIC), Akaike Information Criterion (AIC), and Integrated Completed Likelihood (ICL)—for each tested cluster resolution (K=2:6) using *B-spline* smoothing. Posterior probabilities (mean and median with corresponding standard deviation [SD] or median absolute deviation [MAD]) are included for resolutions where models successfully converged and were selected as optimal in at least one imputed dataset (N=171). Missing values (NA) indicate that the model did not converge, i.e., K-value not selected as the final best fit.

**Supplemental Figure S3: B-spline Smoothed Daily Step Count Trajectories by Cluster**


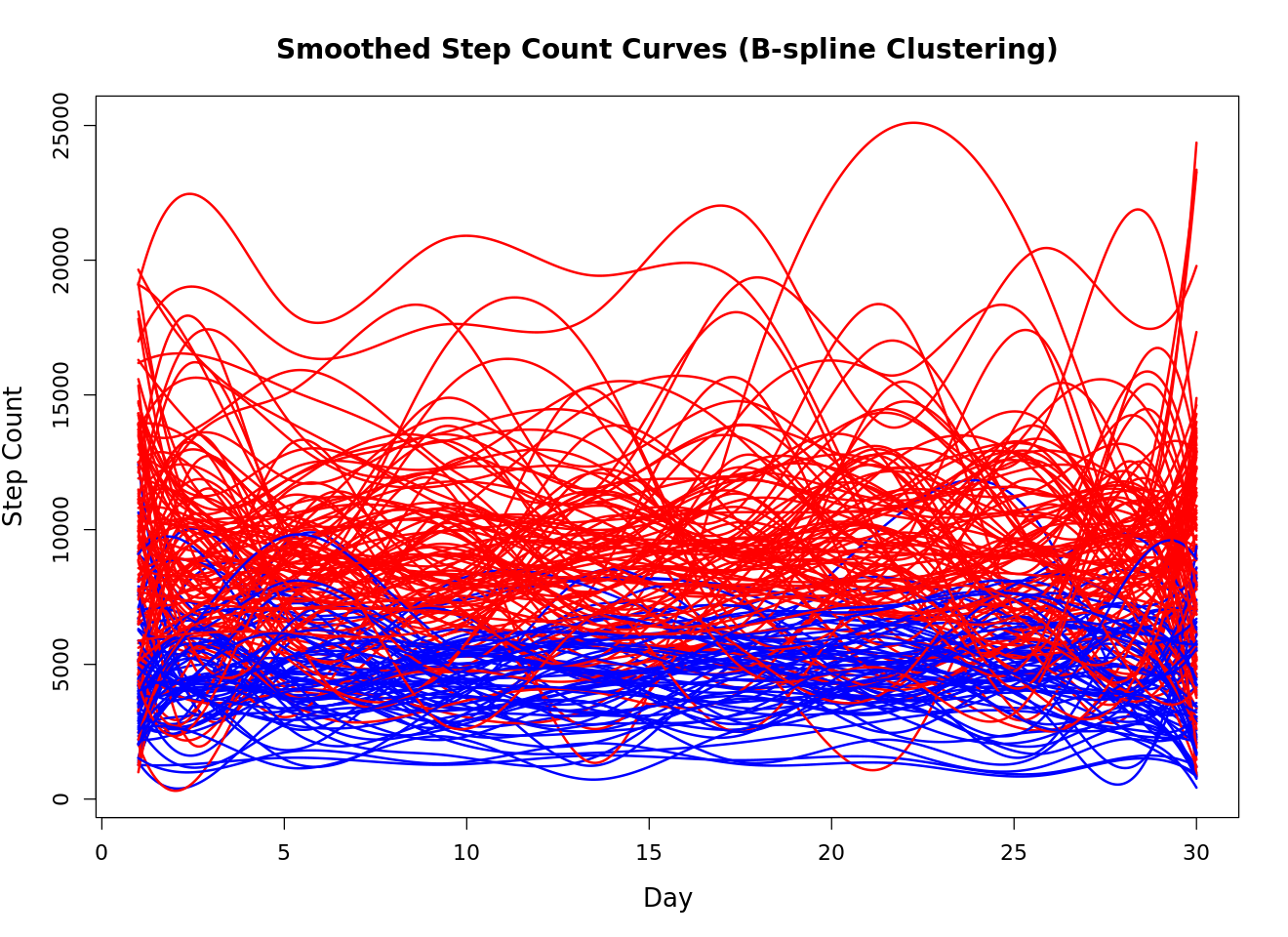


**Supplemental Figure S3:** B-spline smoothed daily step count trajectories on one imputed dataset. The trajectories are color-coded by cluster assignment. Only two clusters were identified, consistent with convergence issues observed in model fitting. Compared to Fourier smoothing, the B-spline approach did not yield clearly separated or interpretable PA phenotypes, further justifying the selection of Fourier basis functions for the final clustering analysis.

**Supplemental Figures S4A-E: Day-Level Cluster Averages of Step Count, Light-, Moderate-, Moderate-to-Vigorous (MVPA)-, and Vigorous Intensity Minutes**

**4A: Day-Level Average Step Counts 4B: Day-Level Average Light Intensity PA**


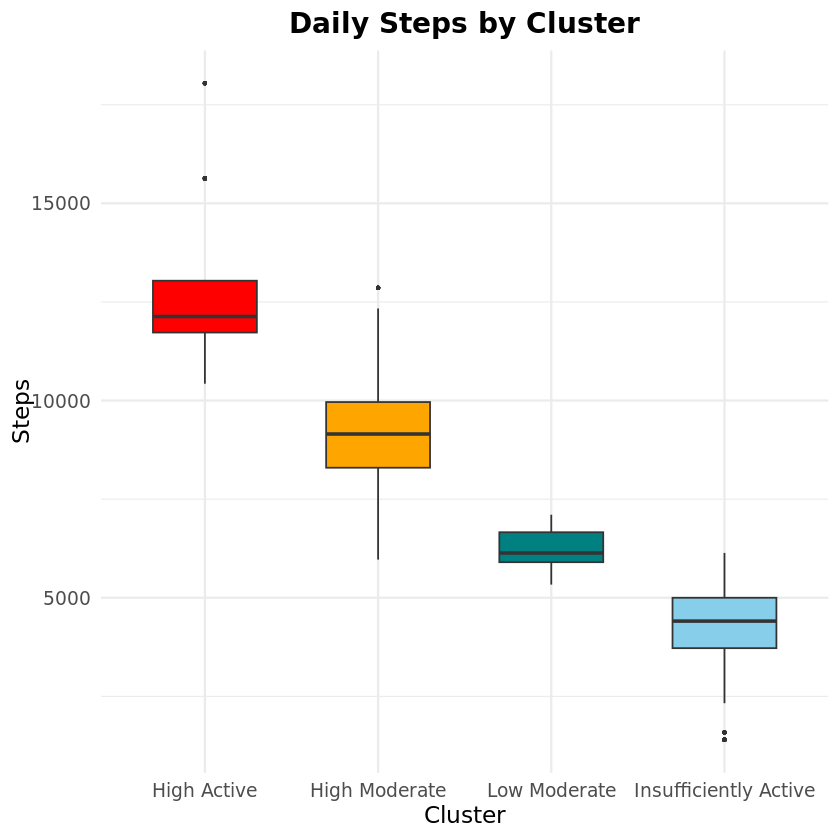

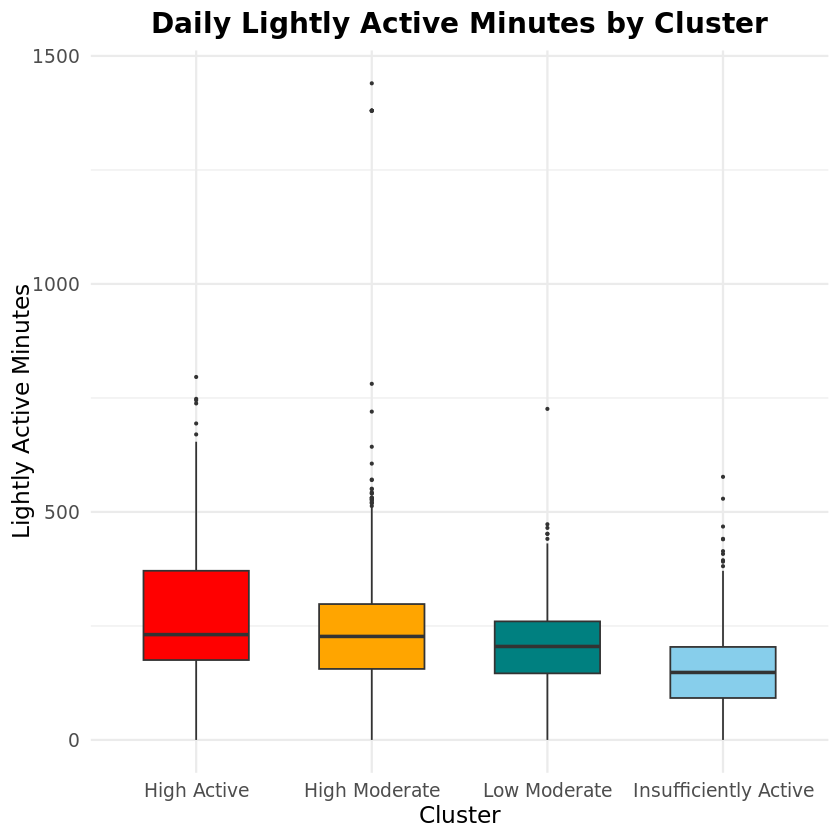


**4C: Day-Level Average Moderate Intensity PA 4D: Day-Level Average MVPA**


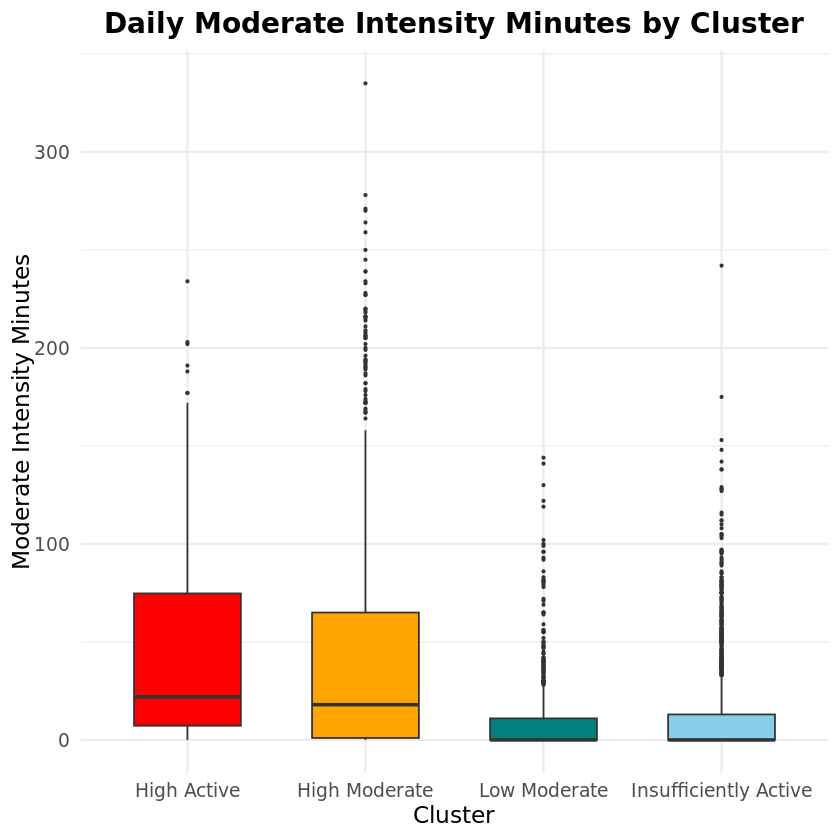
 **
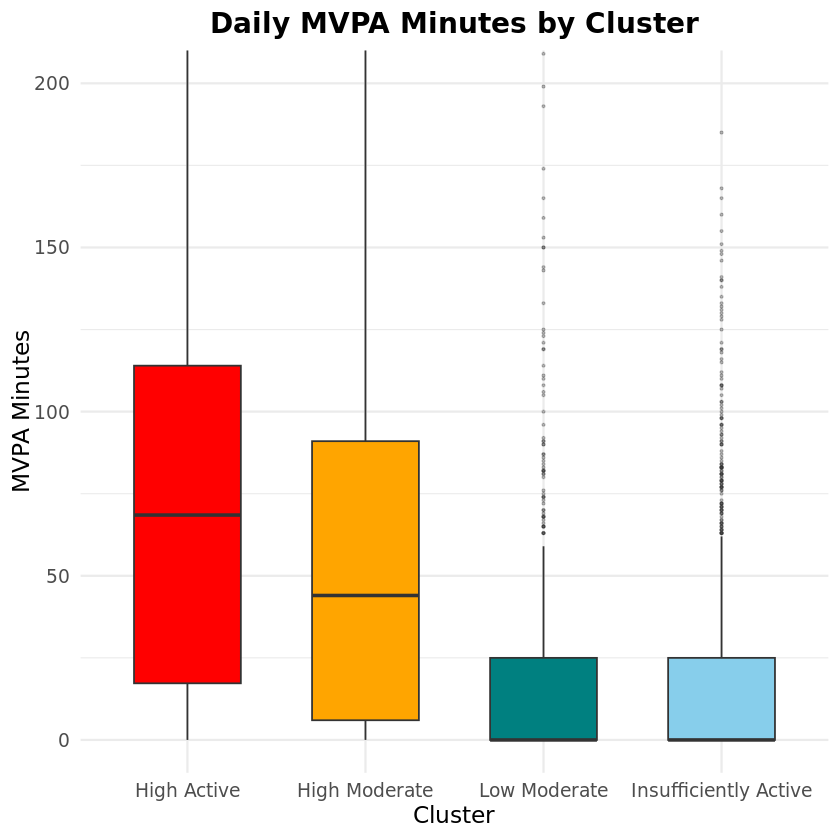
**

**4E: Day-Level Average Vigorous Intensity PA**


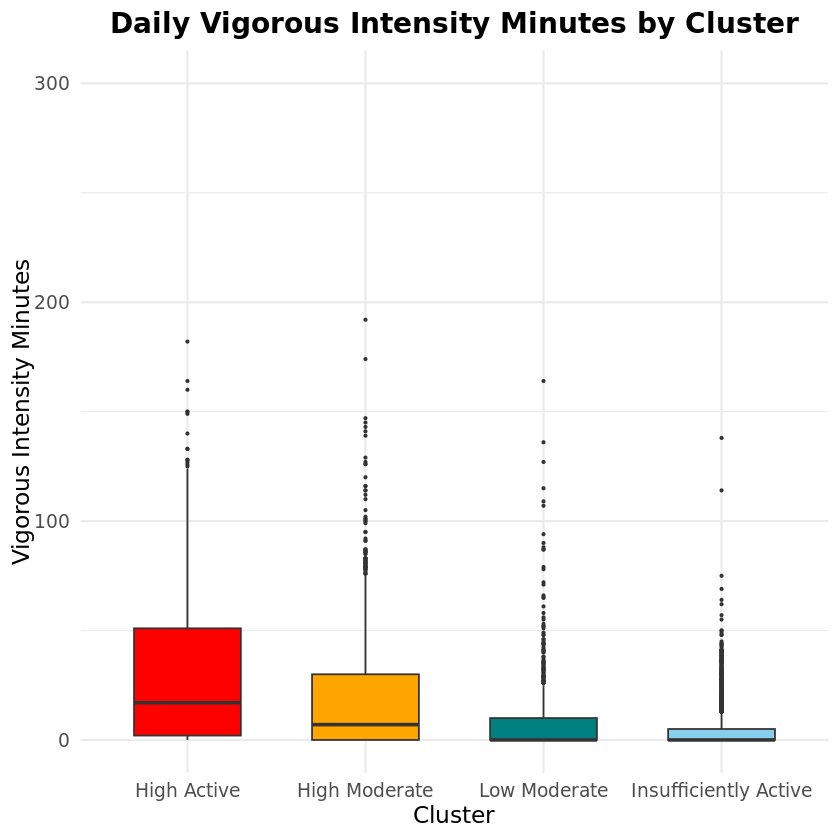


**Supplemental Figure S4 (A-E):** Boxplots of day-level step counts (S4A) and physical activity (PA) minutes of all intensities (light, moderate, moderate-to-vigorous (MVPA), and vigorous – S4B-S4E) across all clusters. Red boxplot indicates the “High Active” cluster, orange boxplot indicates the “High Moderate” cluster, green boxplot indicates the “Low Moderate” cluster, and light blue boxplot indicates the “Insufficiently Active” cluster. Solid black line through boxplots show means for each parameter. Error bars included with each boxplot.

**Supplemental Table S4: Statistical Test Results Comparing PROMIS Pain and Fatigue Across Phenotypes**

| **Test** | **Variable** | **Test Statistic** | **df** | **p-value** | **Pairwise Comparisons** |
| --- | --- | --- | --- | --- | --- |
| Kruskal-Wallis Test | PROMIS Pain Score (continuous) | χ² = 271.8 | 3 | <0.001 | All cluster pairs significantly different (Wilcoxon rank-sum test, Bonferroni-adjusted p < 0.001) |
| Chi-square Test | PROMIS Fatigue Severity (categorical) | χ² = 875.36 | 12 | <0.001 | Descriptive patterns observed (see Figure 5); no post-hoc test applied due to low expected counts in some groups |

**Supplemental Table S4.** *Statistical test results comparing PROMIS pain and fatigue across physical activity phenotypes.* A Kruskal-Wallis test revealed significant differences in PROMIS pain scores across phenotypes, with follow-up Wilcoxon rank-sum tests confirming that each phenotype differed significantly from the others (Bonferroni-adjusted p < 0.001). A Chi-square test also indicated significant differences in the distribution of PROMIS fatigue severity levels across phenotypes. Due to low expected counts in some cells, no pairwise comparisons were conducted for fatigue.

**Supplemental Table S5: Age Summary Statistics by Clustering Phenotype**

| **Phenotype** | **N** | **Mean Age** | **Age SD** | **Min Age** | **Max Age** |
| --- | --- | --- | --- | --- | --- |
| Insufficiently Active | 57 | 61.7 | 16.2 | 31 | 91 |
| High Moderate | 74 | 61.1 | 13.6 | 24 | 82 |
| High Active | 13 | 47.6 | 14.3 | 33 | 73 |
| Low Moderate | 27 | 60.2 | 12.9 | 38 | 82 |

**Supplemental Table S5:** Age characteristics of participants in each of the four identified physical activity phenotypes. Values shown include the number of individuals per cluster (N), as well as the mean, standard deviation (SD), minimum, and maximum age in years.
